# The predictive role of the platelet-to-lymphocyte ratio for the risk of non-alcoholic fatty liver disease and cirrhosis: a nationwide cross-sectional study

**DOI:** 10.3389/fendo.2024.1376894

**Published:** 2024-07-08

**Authors:** Cheng Yan, Weichang Zhang, Yangyan Xiao, Yuxin Sun, Xinke Peng, Wenwu Cai

**Affiliations:** ^1^ Department of General Surgery, Second Xiangya Hospital, Central South University, Changsha, Hunan, China; ^2^ Department of Vascular Surgery, Second Xiangya Hospital, Central South University, Changsha, Hunan, China; ^3^ Department of Ophthalmology, Second Xiangya Hospital, Central South University, Changsha, Hunan, China; ^4^ Department of Rehabilitation, The First Affiliated Hospital of Hengyang Medical School, University of South China, Hengyang, Hunan, China

**Keywords:** NHANES, NAFLD, NASH, platelet-to-lymphocyte ratio, cirrhosis

## Abstract

**Background:**

The associations between platelet-to-lymphocyte ratio (PLR) and non-alcoholic fatty liver disease (NAFLD) and cirrhosis are unclear, and there are still no effective means for diagnosing or monitoring disease progression.

**Methods:**

Data from the National Health and Nutrition Examination Surveys were collected for analysis. Logistic regression and restricted cubic splines were used to evaluate the associations between PLR and NAFLD and cirrhosis in different populations. The Area Under Curve Receiver Operating Characteristic (AUCROC) was used to distinguish the models. Threshold analysis was performed by constructing a two-piecewise linear regression. Correlation analysis was performed separately on either side of the inflection point.

**Results:**

A total of 5724 adults were included. Logistic regression analysis revealed that the PLR was associated with NAFLD and cirrhosis (AUCROC of NAFLD: 0.803; AUCROC of cirrhosis: 0.851). The AUCROC of the PLR for predicting NAFLD incidence was 0.762 in the diabetic population and 0.804 in the nondiabetic population. High PLR predicted cirrhosis in the diabetic population, with an AUCROC of 0.824, whereas a high PLR was not associated with cirrhosis in the nondiabetic population. The restricted cubic spline revealed a negative linear correlation between the PLR and NAFLD incidence. The inflection point of the PLR for NAFLD was 180.74. A PLR ≤180.74 was statistically significant (odds ratio=0.997, 95% confidence interval=0.995-0.999). In the NAFLD population, the PLR was negatively correlated with cirrhosis at a PLR ≤130.5 (odds ratio=0.987, 95% confidence interval=0.977-0.996) and positively correlated with cirrhosis at a PLR > 130.5 (odds ratio=1.006, 95% confidence interval=1.001-1.012).

**Conclusions:**

The PLR and NAFLD were negatively correlated in the U.S. population. The PLR had a U-shaped relationship with cirrhosis in the NAFLD population. The PLR has potential value in monitoring NAFLD patient progression to cirrhosis.

## 1 Introduction

Non-alcoholic fatty liver disease (NAFLD) has become the most common cause of chronic liver disease worldwide. It affects approximately a quarter of the global population, with varying prevalence rates across different regions. These rates range from 13.5% in Africa to 31.8% in the Middle East and more than 34.0% in the United States (1). The prevalence of NAFLD is increasing in Asia (2). NAFLD is characterized by abnormal lipid deposition in the liver and damage to liver cells (3). Several factors, including caloric intake, lifestyle habits, body fat distribution, socioeconomic status, and genetics, can influence the development of NAFLD. The spectrum of NAFLD ranges from simple steatosis to non-alcoholic steatohepatitis (NASH), both of which are symptoms of the disease. Most studies suggest that NASH is a progressive form of NAFLD, and it is often associated with rapid fibrosis progression. However, there is also evidence suggesting that a small percentage of NAFLD patients without histological features of NASH can progress to advanced liver fibrosis and even cirrhosis (4). Advanced liver fibrosis and cirrhosis are important factors contributing to long-term outcomes and mortality (5).

Unfortunately, many NAFLD patients can go undiagnosed for extended periods, and some are diagnosed only when they exhibit symptoms of splenomegaly, thrombocytopenia, portal hypertension, or liver-related complications. A considerable proportion of patients diagnosed with NAFLD experience adverse outcomes, including advanced fibrosis, cirrhosis, and even hepatocellular carcinoma within a span of 10 to 20 years. Therefore, early diagnosis and screening of NAFLD and cirrhosis are crucial for monitoring disease progression (6). Traditionally, as the gold standard for the clinical diagnosis of liver fibrosis, liver biopsy has been used to characterize and quantify the histological features of steatosis and fibrosis. However, the greatest limitation of liver biopsies is that it is an invasive procedure, and thus, they are not suitable for widespread assessment of disease stage or progression (7). As a result, research on non-invasive and effective detection methods for NAFLD and liver fibrosis continues to be carried out, and serum biomarkers are the focus of this field of research. Several studies have explored the potential of inflammatory markers as non-invasive indicators from the perspectives of immunity and inflammation (8). However, the prediction of NAFLD and cirrhosis remains challenging.

The platelet-to-lymphocyte ratio (PLR) is an indicator of inflammation and can be easily obtained from routine blood tests. The PLR has been extensively studied as a prognostic predictor for malignant tumors (9, 10), Additionally, in studies of inflammatory and immune diseases, the PLR has been shown to be correlated with the presence of rheumatoid arthritis (11). However, to our knowledge, no study has examined the relationship between the PLR and NAFLD or cirrhosis incidence. Accordingly, the purpose of this study was to determine the association between the PLR and NAFLD and cirrhosis incidence.

## 2 Materials and methods

### 2.1 Study population

**Figure 1:**
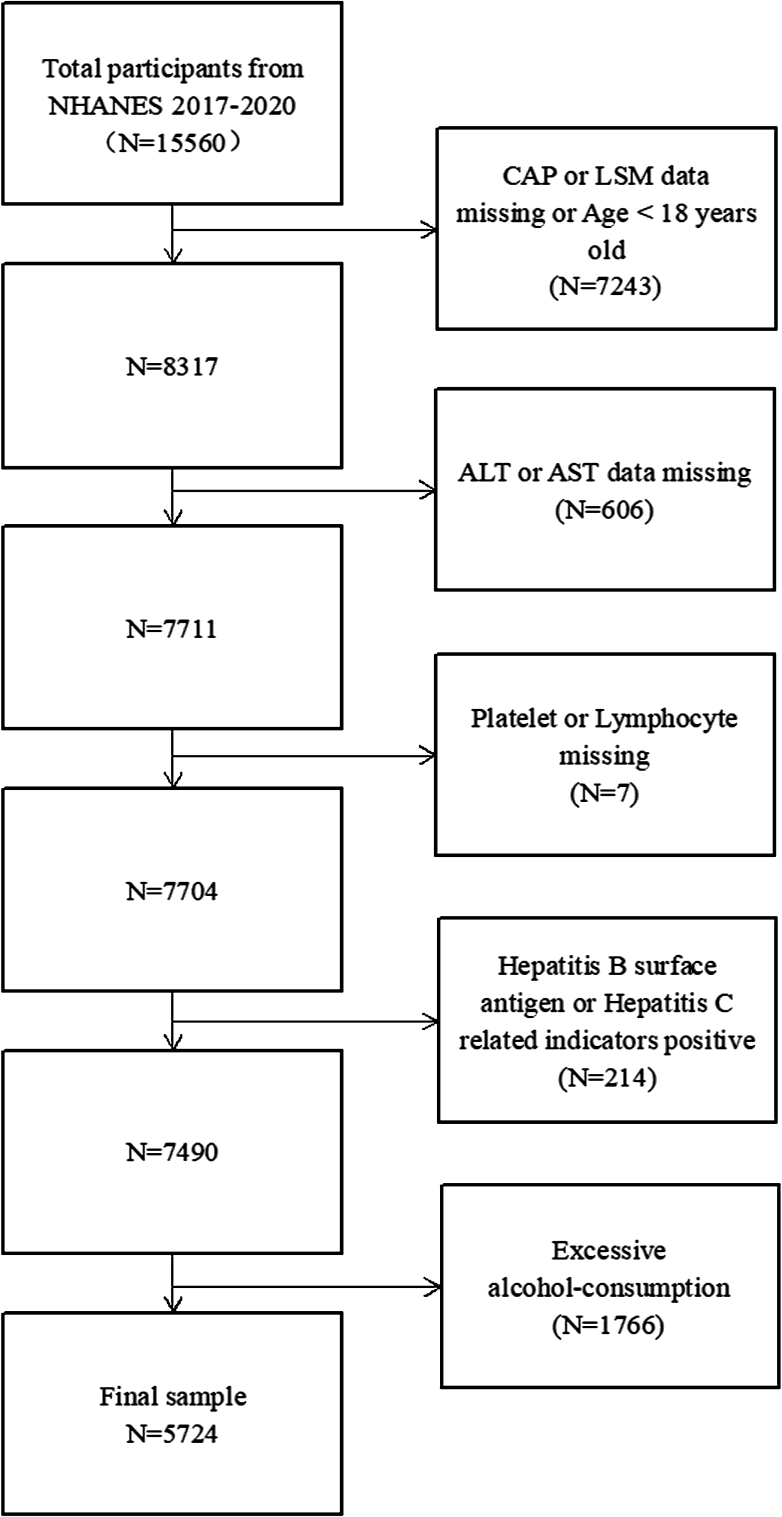
Flow chart of participants selection.

The National Health and Nutrition Examination Survey (NHANES) is a database that records the health and nutritional status of the U.S. population through a complex and multiple-stage probability sampling method. This survey used the 2017–2020 March continuous cycle of the NHANES. We gradually excluded a total of 9836 participants (**Figure 1**). The study ultimately included 5724 participants.

### 2.2 Study variables

Demographic baseline data, including age, sex, race, height, weight, the ratio of family income to poverty, smoking status, and sleeping time, were obtained from the original database. Diabetes history and drug use information were obtained through questionnaires. Laboratory parameters, including white blood cell count, lymphocyte count, monocyte count, hemoglobin, platelet count, alanine aminotransferase (ALT), aspartate aminotransferase (AST), alkaline phosphatase (ALP), gamma-glutamyltransferase (GGT), lactate dehydrogenase (LDH), high-density lipoprotein (HDL), low-density lipoprotein (LDL), and hepatitis-related parameters, were evaluated using standard laboratory methods.

### 2.3 Diagnostic criteria and definitions of groups

The final included population underwent vibration-controlled transient elastography, which can provide a controlled attenuation parameter (CAP) and liver stiffness measurement (LSM). In this study, NAFLD was defined as CAP ≥274 dB/m, and cirrhosis was defined as LSM ≥12 kPa (12, 13). The amount of alcohol consumed is reported in glasses, with one alcoholic beverage defined as 12oz beer, 5oz red wine or 1.5oz white wine, and uniformly converted to grams. For men, ≥30 g/day was considered to indicate excessive drinking, and for women, ≥20 g/day was considered to indicate excessive drinking. Body mass index (BMI) was calculated by dividing the weight in kilograms by the square of the height in meters. According to the ADA’s diagnostic criteria for diabetes (14), diabetes was defined as a self-reported diagnosis, use of insulin or hypoglycemic drugs, fasting glucose ≥126 mg/dL, or glycohemoglobin level ≥6.5%. Fibrosis 4 score (Fib-4) = age × AST/PLT × √ALT. Triglyceride glucose index (TygI) = Ln [triglyceride (mg/dl) × glucose (mg/dl)]/2. In this study, the mean value of the TygI cohort (4.72) was used as the cutoff for dividing the high group and the low group for analysis. The PLR was analyzed in terms of its continuous variable, categorical variable and quartile variable and was divided into high and low groups according to the mean value (125.28). Categorical variables are represented by the PLR (cat). Continuous variables are represented by the PLR (con).

### 2.4 Ethical considerations

The NHANES program was reviewed and approved by the NCHS Research Ethics Review Committee. The original researchers provided participants with a detailed study purpose and methodology and ensured that they understood the content and possible risks of the study. Additionally, all survey participants signed informed consent forms. The NHANES data were deidentified and anonymized during the data analysis, and secondary analysis did not require any additional ethical approval or informed consent.

### 2.5 Statistical analysis

All the statistical analyses were performed with SPSS (version 25.0) and R (version 4.3.1) software (using the “tidyverse” package in R for data processing and the “plotRCS” package for the production of restricted cubic splines). Continuous variables are expressed as the mean ± SD. Categorical variables are expressed as numbers and percentages. T test was used to analyze variables with a normal distribution. The Mann Whitney U test was used to analyze variables with a nonnormal distribution, and the chi square test was used to study the difference in rates between classification groups. Logistic regression analysis was used to evaluate the associations of the PLR, Fib-4 level and TygI with NAFLD and cirrhosis incidence. The diagnostic accuracy, sensitivity and specificity of the different models were evaluated by the area Under Curve Receiver Operating Characteristic (AUCROC). The linear relationship was evaluated by using a restricted cubic spline, and four Knots (0.05, 0.35, 0.65, and 0.95) were selected. Threshold analysis was performed by selecting the highest likelihood by segmented regression. Segmented logistic regression was used on both sides of the inflection point. All tests were two-tailed, and results with p < 0.05 were considered to indicate statistical significance.

## 3 Results

### 3.1 Demographic data

In this study, a total of 5724 adults with an average age of 51.4 ± 18.3 years were included based on the predefined inclusion and exclusion criteria. Among them, 56.1% were female, and 43.9% were male. The sample population was categorized into different groups according to CAP and LSM measurements (**Supplementary Table 1**).

Among participants stratified based on CAP, the mean age, proportion of males, and percentage of non-Hispanic white individuals and Mexican Americans were greater in the NAFLD group. Additionally, compared with the non-NAFLD group, the NAFLD group had a higher BMI, more codiabetic individuals, and a greater incidence of smoking. In terms of laboratory examination results, compared with patients in the non-NAFLD group, patients in the NAFLD group displayed significant differences in white blood cell, lymphocyte, monocyte, and platelet counts and ALT, AST, LDH, GGT, and HDL levels. The LSM values were 5.15 ± 4.15 for the non-NAFLD group and 7.01 ± 6.22 for the NAFLD group. There was no significant difference observed in the ratio of family income to poverty.

Among participants stratified based on LSM, there was a higher proportion of elderly patients, non-Hispanic White individuals, and individuals with codiabetes, greater BMI values, increased white blood cell, monocyte, and platelet counts and elevated levels of ALT, AST, ALP, GGT and LDH in patients with cirrhosis. Additionally, these patients exhibited lower HDL levels. The CAP values were 261 ± 61.4 for the non-NAFLD group and 322 ± 62.6 for the NAFLD group. The ratio of family income to poverty showed no significant difference between the two groups.

### 3.2 Associations of the PLR, Fib-4 and TygI with NAFLD and cirrhosis

**Supplementary Table 2** shows the results of the logistic regression analysis. We analyzed the relationships between the PLR, TygI, or Fib-4 and NAFLD and cirrhosis according to the different models. According to all the models, the PLR was strongly negatively correlated with NAFLD incidence. In Model 3, the risk of NAFLD in the highest quartile decreased by 30.9% compared with that in the lowest quartile (OR=0.691, 95% CI=0.581-0.823). The same trend was observed in patients with diabetes, in which the risk of NAFLD decreased by 47.6% in the highest quartile compared with the lowest quartile (OR=0.524, 95% CI=0.354-0.775). A high PLR was still negatively correlated with the occurrence of NAFLD in patients without diabetes (OR=0.847, 95% CI=0.733-0.978). According to the comparison of the three models, only the second quartile was not significantly correlated with the lowest quartile. In Model 3, the high TygI group was significantly positively correlated with high NAFLD in both the total population and the other subgroups. Fib-4 showed a positive association with NAFLD in both the total population and nondiabetic population (OR: 1.174, 95% CI: 1.066-1.292; OR: 1.148, 95% CI: 1.027-1.283), but the association was not significant in patients with diabetes.

A correlation between the PLR and cirrhosis incidence was observed in multiple models. In the total population, a high PLR was negatively correlated with cirrhosis according to all three models. Among the models, the risk of cirrhosis in the high-PLR group was decreased by 37.1% in Model 3 (OR=0.629, 95% CI=0.460-0.861). According to the interquartile range analysis, the third quartile was significantly negatively correlated with cirrhosis incidence compared with the lowest quartile (OR=0.459, 95% CI=0.303-0.696). In the diabetic population, the high PLR was still negatively correlated with cirrhosis according to all the models, and the risk of cirrhosis in the third quartile was reduced by 76% compared with that in the lowest quartile (OR=0.240, 95% CI=0.114-0.507). However, the correlation was not significant in the second quartile or the highest quartile compared with that in the lowest quartile. In contrast, in patients without diabetes, only the second quartile and the third quartile were significantly correlated with cirrhosis compared with the lowest quartile. The high TygI group was positively correlated with cirrhosis only in Model 1 (OR=2.339, 95% CI=1.577-3.467), and the correlation was not significant after adjusting for variables. Fib-4 values were significantly positively correlated with cirrhosis in all groups.

**Figure 2:**
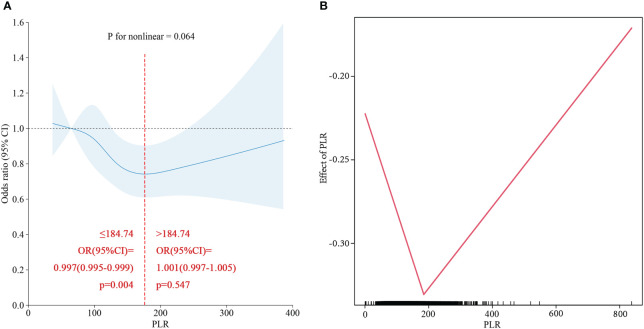
**(A)** Restricted cubic spline of PLR for NAFLD in total population. **(B)** Two-piecewise linear regression of PLR for NAFLD in total population.

To verify the relationship between the PLR and NAFLD incidence, a restricted cubic spline was used. As shown in **Figure 2**, the restricted cubic spline indicated that the relationship between the PLR and NAFLD was linear (p for nonlinear=0.064). Two- piecewise linear regression was used to further estimate the threshold effect, and the inflection point was 180.74. Analysis of both sides of the inflection point indicated that a PLR less than 184.74 was linearly negatively correlated with the risk of NAFLD, while a PLR greater than 184.74 was not significantly correlated with NAFLD risk.

### 3.3 Diagnostic performance of the PLR, TygI, and Fib-4 for NAFLD and cirrhosis

**Figure 3:**
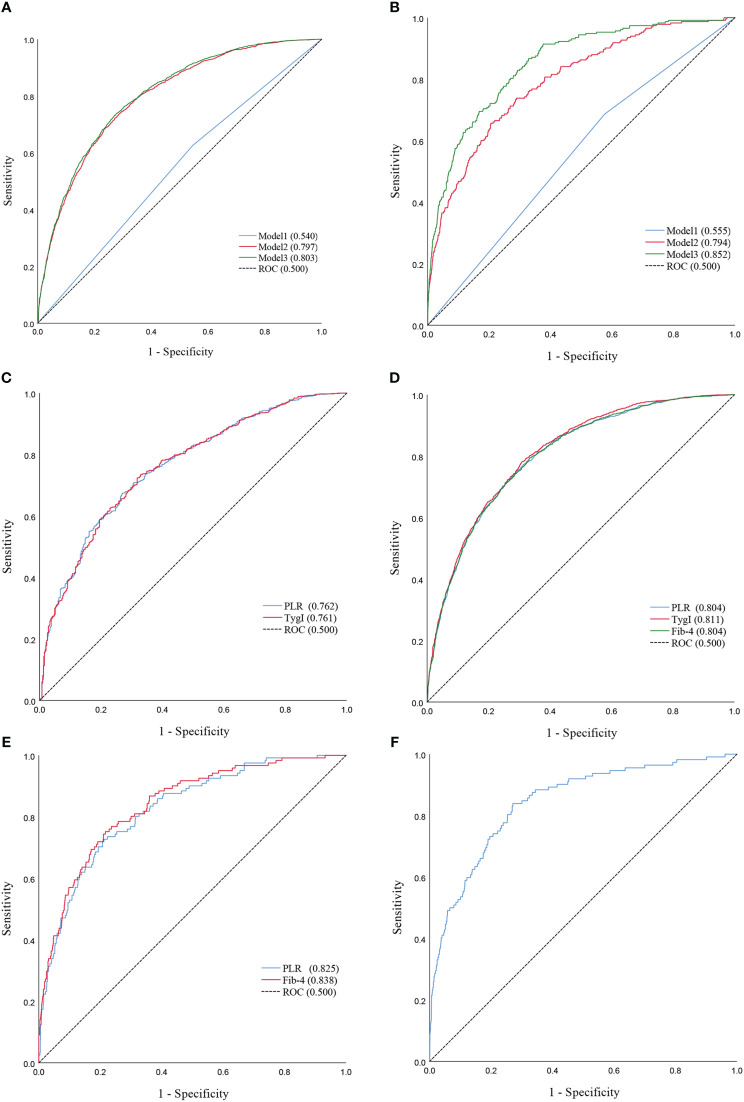
**(A)** ROC curves of Model1 and Model2 and Model3 for NAFLD in the total population. **(B)** ROC curves of Model1 and Model2 and Model3 for Cirrhosis in the total population. **(C)** ROC Curve of PLR and TygI for NAFLD in the diabetic population. **(D)** ROC Curve of PLR and TygI and Fib-4 for NAFLD in the non-diabetic. **(E)** ROC Curve of PLR and Fib-4 for Cirrhosis in the diabetic population. **(F)** ROC Curve of Fib-4 for Cirrhosis in the non-diabetic population.

Combined with the results of the logistic regression analysis, ROC curve analysis was used to evaluate the diagnostic ability and performance of PLR (cat), TygI and Fib-4 among the three models for NAFLD and cirrhosis. The results are shown in **Table 1**; **Figure 3**. In the total population, the PLR AUCROC (95% CI) of Model 1 for NAFLD was 0.540 (0.525-0.555), while the AUCROC (95% CI) of Model 2 and Model 3 for NAFLD were 0.797 (0.786-0.808) and 0.803 (0.792-0.814), respectively. In the diabetic population, the AUCROCs (95% CI) for PLR and TygI for NAFLD were 0.762 (0.734-0.791) and 0.761 (0.733-0.790), respectively. In the nondiabetic population, the AUCROC (95% CI) corresponding to the PLR, TygI and Fib-4 were 0.804 (0.791-0.817), 0.811 (0.798-0.823) and 0.804 (0.791-0.817), respectively. In the total population, the AUCROC (95% CI) of the PLR for cirrhosis in Model 1 and Model 2 were 0.555 (0.519-0.592) and 0.794 (0.765-0.824), respectively, while the AUCROC of Model 3 reached 0.852 (0.826-0.876). The AUCROC (95% CI) of PLR and Fib-4 for cirrhosis were 0.825 (0.786-0.863) and 0.838 (0.801-0.876), respectively, in the diabetic population. Only Fib-4 was associated with cirrhosis in nondiabetic subjects, and the AUCROC (95% CI) was 0.844 (0.806-0.882).

**Table 1:** The ROC curve parameters for the stratified population.

		AUCROC	95%CI	Youden index	Specificity	Sensitivity
**A**	**PLR**	0.762	0.734-0.791	0.403	0.729	0.674
	**TygI**	0.761	0.733-0.790	0.407	0.672	0.735
**B**	**PLR**	0.804	0.791-0.817	0.460	0.713	0.747
	**TygI**	0.811	0.798-0.823	0.473	0.690	0.783
	**Fib-4**	0.804	0.791-0.817	0.459	0.662	0.797
**C**	**PLR**	0.825	0.786-0.863	0.515	0.788	0.727
	**Fib-4**	0.838	0.801-0.876	0.535	0.783	0.752
**D**	**Fib-4**	0.844	0.806-0.882	0.568	0.729	0.839

A, ROC Curve of PLR and TygI for NAFLD in the diabetic population; B, ROC Curve of PLR and TygI and Fib-4 for NAFLD in the non-diabetic; C, ROC Curve of PLR and Fib-4 for Cirrhosis in the diabetic population; D, ROC Curve of Fib-4 for Cirrhosis in the non-diabetic population.

### 3.4 Relationships between the PLR and the CAP and LSM in different populations

**Figure 4:**
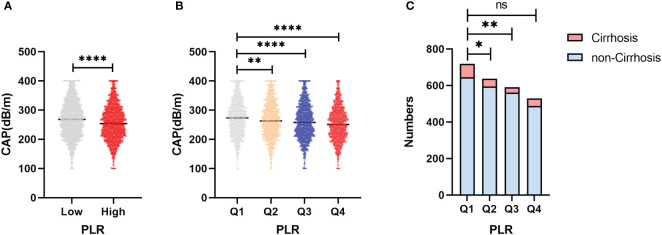
**(A)** Differences in CAP between the high PLR group and the low group. **(B)** Differences in CAP between the PLR quartile groups. **(C)** Differences in the distribution among PLR quartiles groups of patients progressing to cirrhosis in NAFLD patients. *p<0.05, **p<0.01, ****p<0.0001.

**Figure 5:**
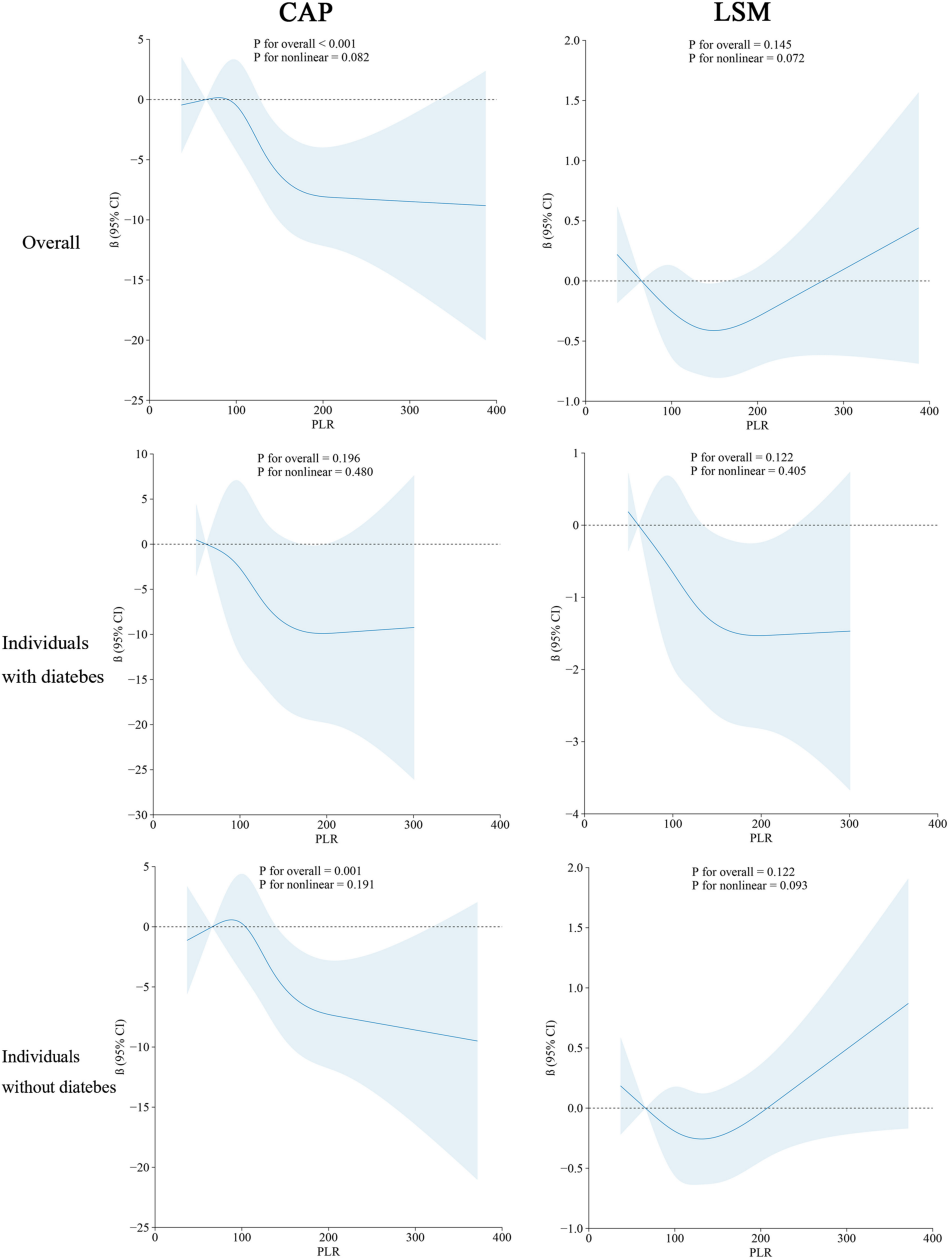
Restricted cubic spline of PLR and CAP/LSM in the different population.

**Figures 4A, B** shows the distribution of CAP between the high-PLR group, low-PLR group and quartile groups. There was a statistically significant difference between the high-PLR group and the low-PLR group, and the CAP values of the second, third and highest quartiles were also significantly different from those of the lowest quartile. We determined whether there was a linear correlation between the PLR and CAP, and then we used a restricted cubic spline to analyze the relationship between the PLR and CAP in different populations (**Figure 5**). In the total population and the nondiabetic population, the PLR showed a linear relationship with the CAP (P for nonlinear = 0.082, P for nonlinear =0.191) after adjusting for variables, but the relationship was not significant in the diabetic population. The restricted cubic spline analysis of the PLR and LSM was also performed. However, no correlation was found between the PLR and LSM in the total population, diabetic population or nondiabetic population.

### 3.5 Associations between the PLR and cirrhosis in the NAFLD population

**Figure 6:**
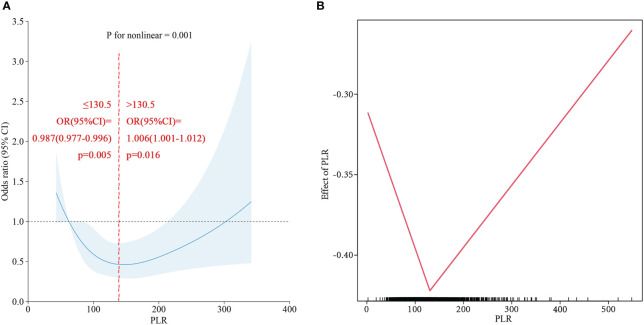
**(A)** Restricted cubic spline of PLR for Cirrhosis in NAFLD population. **(B)** Two-piecewise linear regression of PLR for Cirrhosis in NAFLD population.

Based on the PLR quartile, we found that the number of patients with cirrhosis in the second quartile and the third quartile significantly differed from the number of patients with cirrhosis in the lowest quartile in the NAFLD population (**Figure 4C**); however, there was no significant difference between the highest and lowest quartile groups. The results of the restricted cubic spline suggested that the PLR had a U-shaped relationship with cirrhosis in the NAFLD population (p for nonlinear =0.001) (**Figure 6**). The best inflection point is 130.5. In the NAFLD population, a PLR less than 130.5 was negatively correlated with cirrhosis (OR=0.987, 95% CI=0.977-0.996), and a PLR above 130.5 was positively correlated with cirrhosis (OR=1.006, 95% 1.001-1.012).

## 4 Discussion

The objective of this study was to explore the potential value of the PLR in predicting NAFLD and cirrhosis. Platelets, which are functional and non-nucleated immune cells, play crucial roles in coordinating both innate and acquired immune responses through various mechanisms, such as complement activation, immune complex perception, T-cell activation, and dendritic cell activation (15). Moreover, lymphocytes are closely associated with inflammatory function. The activation of proinflammatory M1 macrophages leads to the production of cytokines that recruit proinflammatory cells, including innate immune cells and T lymphocytes, thereby initiating an inflammatory cascade (16). Therefore, investigating the PLR can provide valuable insights into the inflammatory immune response. Objectively, numerous experiments and analyses have produced positive results. In infectious and inflammatory diseases, the PLR has been shown to have predictive value for sepsis mortality (17), and the PLR also plays a role in the progression and prognosis of chronic liver inflammation. In a retrospective study involving 184 patients (18), a significant reduction in the PLR was found in patients with HCV-associated cirrhosis and HCV-associated hepatocellular carcinoma by analyzing HCV-infected patients with different stages of liver disease and chronic hepatitis C patients with different virological responses after treatment. The PLR decreases with the incidence of HCV infection-associated liver disease. In addition, the PLR was compared with the neutrophil-to-lymphocyte ratio (NLR), another serum inflammatory index, reflecting the preference for PLR over NLR in disease prediction. The study by Cucoranu DC (19) also failed to identify a correlation between the NLR and hepatic steatosis by assessing the liver attenuation value. However, NLR did demonstrate a favorable performance in predicting alcoholic cirrhosis (AUCROC = 0.821) (20). These findings cast the role of the NLR in a controversial light and guide our selection of research direction. Our study further describes the new application of PLR.

NAFLD is defined as the presence of steatosis in more than 5% of liver cells and the presence of metabolic risk factors (particularly obesity and type 2 diabetes) without excessive alcohol consumption or other chronic liver diseases (21). NASH, which is often associated with rapid liver fibrosis, is considered a progressive form of NAFLD. According to a cohort study in which patients were followed for more than 10 years (22), more than ten percent of NASH patients developed end-stage liver disease. The “three-stroke” process has been used to describe the pathological progression of NAFLD, namely, steatosis, lipotoxicity, and inflammation (23). Hepatic inflammation is an important driving force for the progression of NAFLD and NASH. Luo Y (24)demonstrated that the inflammatory pathway plays a key role in the development of NAFLD. Once inflammation is triggered in NAFLD, it will continue through the vicious cycle of steatosis, lipotoxicity and inflammation, and continuous liver fibrosis will eventually lead to cirrhosis and even liver cancer. In this study, an increased PLR was associated with a decreased risk of NAFLD (AUCROC=0.803). Interestingly, our observations revealed that PLR exhibited higher diagnostic accuracy for NAFLD among the non-diabetic population compared to the diabetic population (AUCROC=0.804/AUCROC=0.762). Additionally, PLR was found to be associated with NAFLD specifically within the non-obese population (25), aligning with the observation (26) that the risk of detecting NAFLD in patients with obstructive sleep apnea–hypopnea syndrome via PLR was more favorable in those with a BMI <28 kg/m². This suggests that the non-metabolic disorders population may be more appropriate for the application of PLR. Furthermore, the study has explored the combination of PLR and the white blood cell to mean platelet volume ratio to enhance diagnostic efficacy, which may represent a promising direction for future research.

Overnutrition is another driver of NAFLD that leads to insulin resistance (27). Insulin resistance has long been recognized as a risk factor for steatosis. In defining the causative drivers of simple steatosis and NASH, it is generally accepted that the reduced metabolic capacity of the liver leads to the accumulation of toxic lipid substances. Steatosis and fat infiltration caused by insulin resistance are both causes and consequences of this disease. Therefore, TygI, a biological marker of insulin resistance, was selected for analysis to assess insulin resistance (28). TygI had a high accuracy (AUCROC=0.858) in the diagnosis of insulin resistance, with a sensitivity and specificity of 96.5% and 85.0%, respectively (29). In our study, TygI was significantly positively correlated with the incidence of NAFLD, which confirmed that insulin resistance is an important factor in the occurrence and development of NAFLD. However, the association between TygI and cirrhosis was not significant. It is well known that portal shunts caused by cirrhosis are also an important cause of insulin resistance. Shunting of portal-hypertensive-associated insulin from the portal venous system into the systemic circulation causes hyperinsulinemia, insulin receptor desensitization and insulin downregulation, leading to insulin resistance (30). However, the results of this study may suggest the importance of multiple factors in the outcome of cirrhosis, and insulin resistance does not seem to be an important feature in the identification of cirrhosis. Fib-4, an indicator of liver fibrosis, is regarded as an important auxiliary tool in the primary clinical system. Liver fibrosis is the most important risk factor for liver cancer in patients with NAFLD and decompensated cirrhosis (31). Previous studies (32) indicated that the risk of liver-related events in the Fib-4>2.67 subgroup was greater than that in the Fib-4<1.30 subgroup (95% CI: 13.1-14.6). A large cohort study involving 11154 participants who were followed for 14.5 years also showed that advanced liver fibrosis detected with a Fib-4 > 2.67 was a predictor of mortality (adjusted hazard ratio: 1.66) (33). Shah (34)analyzed the value of Fib-4 in the diagnosis of NAFLD-associated fibrosis in 541 patients, with an overall accuracy of 89%. In this study, we found that Fib-4 could predict cirrhosis well in both people with and without diabetes (AUCROC: 0.839 and 0.841), and it was better than the prediction accuracy of KM’s study for advanced liver fibrosis in obese people (AUCROC: 0.74) (35). Although the sensitivity can reach 0.830 in people without diabetes, unfortunately, Fib-4 has a limited ability to predict NAFLD. Fib-4 was positively correlated with NAFLD in the total population but was not significantly correlated with NAFLD in the diabetic population after stratification,while it was correlated with NAFLD in the nondiabetic population, with a specificity of only 0.662. We determined that the severity and stage of NAFLD may be responsible for the decreased ability of Fib-4 to predict NAFLD. As NASH progresses to complete cirrhosis, some histological features may be lost (36). Moreover, staging or stratification of NAFLD patients will have a positive impact on research. Therefore, according to the findings of the present study, the PLR, which is associated with both NAFLD and cirrhosis, could be widely applied at primary hospitals.

One study (37) showed that the CAP accurately predicted steatosis ≥11%, 33%, and 66%, with AUCROC of 0.91, 0.95, and 0.89, respectively. The correlation between CAP and steatosis was also excellent histologically (38). We demonstrated the negative correlation between the PLR and CAP through the restricted cubic spline, which indicated that there was a correlation between the PLR and steatosis. This analysis also confirmed the accuracy of the PLR in predicting NAFLD from another perspective, as excess cholesterol and triglyceride accumulation drive NAFLD progression through direct hepatotoxic effects or postmetabolic lipotoxic metabolites (39).

In clinical work, the identification and monitoring of patients with NAFLD who are at risk of progression to cirrhosis are important issues. Early identification and diagnosis of NASH are priorities for reducing adverse outcomes. According to the study by Zhou (40), there was a negative correlation with NAFLD when the PLR was ≥42.29 (β=0.99, CI=0.98~0.99), and no correlation was detected when the PLR was <42.29, which is similar to the results of our study. In our study, a PLR ≤ 184.74 was negatively linearly correlated with NAFLD incidence, while the number of people with a PLR ≤42.29 accounted for only 0.3% of the total study population. In the future, a larger population stratification analysis may be needed to draw more reliable conclusions. Peter J. Eddowes (41) reported that the degree of steatosis and inflammation were not correlated with the difference in LSM. However, Our study revealed differences in PLR quartiles among patients with cirrhosis in the NAFLD population and further revealed a nonlinear relationship between the PLR and cirrhosis incidence. According to the correlation between the PLR and NAFLD incidence in this study, in patients with a PLR below 130.5, the possibility of disease progression gradually increased with decreasing PLR. In patients with a PLR greater than 130.5, as the PLR gradually increased, the risk of cirrhosis also increased. This may verify that patients with NASH may have little to no steatosis (42). This result also has potential implications for the identification and monitoring of cirrhosis patients in the NAFLD population.

To our knowledge, this is the first population-based study linking the PLR to NAFLD and cirrhosis. Three noninvasive scoring systems were used to assess three-way associations, and the PLR was strongly associated with NAFLD and cirrhosis. This study has several limitations. First, CAP and LSM were used to group disease groups and control groups. Despite their good accuracy, liver biopsy is still the gold standard for diagnosis, and the difficulty in obtaining samples limits the development of related research. Second, this was an analytical study based on the American population. Due to the multifactorial nature of NAFLD, its generalizability may be limited to other ethnic groups. Moreover, prospective studies with large sample sizes are still necessary to avoid the impact of possible confounding factors.

## 5 Conclusions

In the U.S. population, a PLR less than 184.74 was negatively associated with NAFLD, and TygI and Fib-4 were positively associated with NAFLD and cirrhosis, respectively. There was a U-shaped relationship between the PLR and cirrhosis in the NAFLD population. In NAFLD patients with a PLR less than 130.5, the risk of cirrhosis increases with decreasing PLR. In NAFLD patients with a PLR greater than 130.5, the risk of liver cirrhosis increased with increasing PLR. The PLR has potential value in monitoring NAFLD patients’ progression to cirrhosis.

## Data availability statement

The datasets presented in this study can be found in online repositories. The names of the repository/repositories and accession number(s) can be found below: https://www.cdc.gov/nchs/nhanes/index.htm.

## Ethics statement

The studies involving humans were approved by NCHS Research Ethics Review Board. The studies were conducted in accordance with the local legislation and institutional requirements. Written informed consent for participation was not required from the participants or the participants’ legal guardians/next of kin in accordance with the national legislation and institutional requirements.

## Author contributions

CY: Conceptualization, Data curation, Formal analysis, Methodology, Software, Visualization, Writing – original draft. WZ: Formal analysis, Software, Writing – review & editing. YX: Methodology, Writing – review & editing. YS: Data curation, Writing – review & editing. XP: Data curation, Methodology, Writing – review & editing. WC: Conceptualization, Supervision, Writing – review & editing.
