# Supplementary material for: The predictive role of the platelet-to-lymphocyte ratio for the risk of non-alcoholic fatty liver disease and cirrhosis: a nationwide cross-sectional study
Source: Front Endocrinol (Lausanne). 2024 Jul 8;15:1376894. doi: 10.3389/fendo.2024.1376894 (PMC11260703; doi:10.3389/fendo.2024.1376894)
Supplement: Supplementary file 1 [file DataSheet_1.docx]

Supplementary Material

# Supplementary Tables

**Supplementary Table 1**. Characteristics of the study population (N =5724).

| **Variables** | **Non-NAFLD**  **(N=3249)** | **NAFLD**  **(N=2475)** | **P-value** | **Non-cirrhosis**  **(N=5491)** | **cirrhosis**  **(N=233)** | **P-value** | **Total**  **(N=5724)** |
| --- | --- | --- | --- | --- | --- | --- | --- |
| **Age(years)** | 49.0±19.3 | 54.6±16.4 | **＜0.001** | 51.2±18.3 | 56.8±16.4 | **＜0.001** | 51.4±18.3 |
| **Gender** |  |  | **＜0.001** |  |  | **0.001** |  |
| Female | 1956(60.2%) | 1256(50.7%) |  | 3106(56.6%) | 106(45.5%) |  | 3212(56.1%) |
| Male | 1293(39.8%) | 1219(49.3%) |  | 2385(43.4%) | 127(54.5%) |  | 2512(43.9%) |
| **Race/Ethnicity** |  |  | **＜0.001** |  |  | 0.085 |  |
| Mexican American | 280(8.6%) | 357(14.4%) |  | 608(11.1%) | 29(12.4%) |  | 637(11.1%) |
| Non-Hispanic Black | 936(28.8%) | 534(21.6%) |  | 1416(25.8%) | 54(23.2%) |  | 1470(25.7%) |
| Non-Hispanic White | 1091(33.6%) | 917(37.1%) |  | 1911(34.8%) | 97(41.6%) |  | 2008(35.1%) |
| Other Hispanic | 327(10.1%) | 249(10.1%) |  | 552(10.1%) | 24(10.3%) |  | 576(10.1%) |
| Other Race-Including Multi-Racial | 615(18.9%) | 418(16.9%) |  | 1004(18.3%) | 29(12.4%) |  | 1033(18.0%) |
| **Ratio of family income to poverty** | 2.65±1.55 | 2.67±1.51 | 0.536 | 2.66±1.54 | 2.51±1.47 | 0.301 | 2.65±1.54 |
| **BMI(kg/m^2^)** | 27.0±5.95 | 33.6±7.62 | **＜0.001** | 29.5±7.06 | 38.9±10.6 | **＜0.001** | 29.8±7.47 |
| **Diatebes** |  |  | **＜0.001** |  |  | **＜0.001** |  |
| NO | 2949(90.8%) | 1884(76.1%) |  | 4692(85.4%) | 141(60.5%) |  | 4833(84.4%) |
| Yes | 300(9.2%) | 591(23.9%) |  | 799(14.6%) | 92(39.5%) |  | 891(15.6%) |
| **Glycohemoglobin** | 5.63±0.85 | 6.19±1.30 | **＜0.001** | 5.85±1.08 | 6.45±1.42 | **＜0.001** | 5.87±1.11 |
| **Fasting Glucose(mmol/L)** | 5.86±1.45 | 6.82±2.23 | **＜0.001** | 6.23±1.84 | 7.32±2.65 | **＜0.001** | 6.27±1.89 |
| 1. **Reactive Protein(mg/L)** | 3.33±8.22 | 5.20±8.50 | **＜0.001** | 4.03±8.31 | 6.78±9.75 | **＜0.001** | 4.14±8.39 |
| **White blood cell**  **Count(10^9^/L)** | 6.95±7.32 | 7.48±2.11 | **＜0.001** | 7.16±5.80 | 7.54±2.26 | **＜0.001** | 7.18±5.70 |
| **Lymphocyte(10^9^/L)** | 2.22±6.43 | 2.28±0.82 | **＜0.001** | 2.25±4.97 | 2.14±0.88 | 0.425 | 2.24±4.87 |
| **Monocyte(10^9^/L)** | 0.54±0.19 | 0.591±0.226 | **＜0.001** | 0.56±0.21 | 0.62±0.21 | **＜0.001** | 0.56±0.21 |
| **Hemoglobin(g/dL)** | 13.7±1.50 | 14.1±1.55 | **＜0.001** | 13.9±1.53 | 13.8±1.57 | 0.871 | 13.9±1.53 |
| **Platelet(10^9^/L)** | 245±65.0 | 250±66.7 | **0.002** | 248±65.3 | 225±74.1 | **＜0.001** | 247±65.8 |
| **ALT(U/L)** | 17.8±13.4 | 24.7±17.2 | **＜0.001** | 20.4±14.6 | 30.2±27.8 | **＜0.001** | 20.8±15.5 |
| **AST(U/L)** | 20.1±10.7 | 22.0±12.6 | **＜0.001** | 20.5±9.50 | 30.6±32.8 | **＜0.001** | 20.9±11.6 |
| **Albumin(g/L)** | 40.8±3.36 | 40.3±3.24 | **＜0.001** | 40.7±3.28 | 39.0±3.65 | **＜0.001** | 40.6±3.32 |
| **ALP(IU/L)** | 75.8±25.0 | 81.6±25.0 | **＜0.001** | 77.8±24.5 | 90.4±35.5 | **＜0.001** | 78.3±25.2 |
| **GGT(IU/L)** | 24.3±50.2 | 34.1±41.1 | **＜0.001** | 26.9±31.1 | 67.6±171 | **＜0.001** | 28.5±46.7 |
| **LDH(IU/L)** | 158±34.9 | 161±33.5 | **0.004** | 158±32.7 | 177±58.4 | **＜0.001** | 159±34.3 |
| **Total Bilirubin(umol/L)** | 7.93±4.95 | 7.63±4.39 | 0.301 | 7.74±4.65 | 9.28±6.02 | **＜0.001** | 7.80±4.72 |
| **Cholesterol(mmol/L)** | 4.75±1.03 | 4.84±1.06 | **＜0.001** | 4.80±1.04 | 4.47±1.05 | **＜0.001** | 4.79±1.04 |
| **Triglyceride(mmol/L)** | 1.26±0.78 | 1.86±1.25 | **＜0.001** | 1.51±1.05 | 1.74±1.00 | **＜0.001** | 1.52±1.05 |
| **HDL(mmol/L)** | 1.47±0.40 | 1.26±0.35 | **＜0.001** | 1.39±0.39 | 1.24±0.40 | **＜0.001** | 1.38±0.39 |
| **LDL(mmol/L)** | 2.80±0.87 | 2.84±0.91 | 0.098 | 2.83±0.89 | 2.84±0.87 | **＜0.001** | 2.82±0.87 |
| **Blood Urea Nitrogen**  **(mmol/L)** | 5.25±2.05 | 5.55±2.26 | **＜0.001** | 5.34±2.08 | 6.23±3.20 | **＜0.001** | 5.38±2.15 |
| **Creatinine(umol/L)** | 78.5±37.0 | 80.5±47.6 | 0.050 | 79.0±40.2 | 89.0±71.4 | 0.178 | 79.4±41.9 |
| **Sleeping time(hours)** | 7.69±1.67 | 7.49±1.61 | **＜0.001** | 7.60±1.64 | 7.58±1.77 | 0.87 | 7.60±1.65 |
| **Smoked at least 100 cigarettes** |  |  | **0.001** |  |  | 0.091 |  |
| No | 2164(66.6%) | 1547(62.5%) |  | 3572(65.1%) | 139(59.7%) |  | 3711(64.8%) |
| Yes | 1085(33.4%) | 928(37.5%) |  | 1919(34.9%) | 94(40.3%) |  | 2013(35.2%) |
| **LSM(kpa)** | 5.15±4.15 | 7.01±6.22 | **＜0.001** | / | / | / | 5.95±5.23 |
| **CAP(dB/m)** | / | / | / | 261±61.4 | 322±62.6 | **＜0.001** | 264±62.6 |
| **Fib-4** | 1.10±0.75 | 1.13±1.02 | **0.004** | 1.09±0.83 | 1.71±1.54 | **0.004** | 1.11±0.88 |
| **PLR(cat)** |  |  | **＜0.001** |  |  | **0.001** |  |
| Low | 1775(54.6%) | 1550(62.6%) |  | 3165(57.6%) | 160(68.7%) |  | 3325(58.1%) |
| High | 1474(45.4%) | 925(37.4%) |  | 2326(42.4%) | 73(31.3%) |  | 2399(41.9%) |
| **PLR(con)** | 128.98±50.71 | 120.43±48.49 | **＜0.001** | 125.60±49.76 | 117.76±53.60 | **0.019** | 125.28±49.94 |
| **TygI** |  |  | **＜0.001** |  |  | **＜0.001** |  |
| Low | 1146(35.3%) | 253(10.2%) |  | 1370(24.9%) | 29(12.4%) |  | 1399(24.4%) |
| High | 2103(64.7) | 2222(89.8%) |  | 4121(75.1%) | 204(87.6%) |  | 4325(75.6%) |

Supplementary Table 2. Logisitic regression of PLR,TygI,Fib-4 for NAFLD/Cirrhosis in different populations

|  | **NAFLD** | | | | | | **Cirrhosis** | | | | | |
| --- | --- | --- | --- | --- | --- | --- | --- | --- | --- | --- | --- | --- |
|  | **Model1** | | **Model2** | | **Model3** | | **Model1** | | **Model2** | | **Model3** | |
| Overall | OR  (95%CI) | P-value | OR  (95%CI) | P-value | OR  (95%CI) | P-value | OR  (95%CI) | P-value | OR  (95%CI) | P-value | OR  (95%CI) | P-value |
| **PLR**  **(Cat)** | 0.719  (0.646-0.800) | **＜0.001** | 0.787  (0.695-0.891) | **＜0.001** | 0.789  (0.697-0.895) | **＜0.001** | 0.621  (0.468-0.823) | **0.001** | 0.671  (0.497-0.907) | **0.009** | 0.629  (0.460-0.861) | **0.004** |
| **PLR**  **(Con)** | 0.996  (0.995-0.998) | **＜0.001** | 0.998  (0.997-0.999) | **0.001** | 0.998  (0.997-0.999) | **0.001** | 0.996  (0.993-0.999) | **0.018** | 0.998  (0.994-1.001) | 0.132 | 0.997  (0.994-1.000) | 0.082 |
| Q1 |  |  |  |  |  |  |  |  |  |  |  |  |
| Q2 | 0.807  (0.697-0.935) | **0.004** | 0.794  (0.670-0.940) | **0.008** | 0.820  (0.691-0.972) | **0.022** | 0.618  (0.436-0.875) | **0.007** | 0.587  (0.406-0.848) | **0.005** | 0.645  (0.440-0.943) | **0.024** |
| Q3 | 0.704  (0.607-0.815) | **＜0.001** | 0.717  (0.605-0.850) | **＜0.001** | 0.728  (0.614-0.864) | **＜0.001** | 0.486  (0.335-0.707) | **＜0.001** | 0.478  (0.321-0.712) | **＜0.001** | 0.459  (0.303-0.696) | **＜0.001** |
| Q4 | 0.586  (0.505-0.681) | **＜0.001** | 0.675  (0.568-0.802) | **＜0.001** | 0.691  (0.581-0.823) | **＜0.001** | 0.568  (0.398-0.812) | **0.002** | 0.631  (0.430-0.924) | **0.018** | 0.618  (0.415-0.920) | **0.018** |
| **TygI** | 4.786  (4.125-5.553) | **＜0.001** | 3.100  (2.616-3.673) | **＜0.001** | 2.936  (2.474-3.485) | **＜0.001** | 2.339  (1.577-3.467) | **＜0.001** | 1.346  (0.875-2.072) | 0.176 | 0.634  (0.714-1.739) | 0.634 |
| **Fib-4** | 1.043  (0.980-1.111) | 0.184 | 1.183  (1.087-1.287) | **＜0.001** | 1.174  (1.066-1.292) | **0.001** | 1.704  (1.507-1.928) | **＜0.001** | 2.073  (1.798-2.391) | **＜0.001** | 1.596  (1.350-1.888) | **＜0.001** |
| With Diatebes |  |  |  |  |  |  |  |  |  |  |  |  |
| **PLR**  **(Cat)** | 0.618  (0.482-0.792) | **＜0.001** | 0.649  (0.494-0.852) | **0.002** | 0.672  (0.509-0.888) | **0.005** | 0.541  (0.354-0.829) | **0.005** | 0.582  (0.372-0.909) | **0.017** | 0.558  (0.344-0.904) | **0.018** |
| **PLR (Con)** | 0.995  (0.993-0.997) | **＜0.001** | 0.996  (0.994-0.999) | **0.002** | 0.997  (0.994-0.999) | **0.009** | 0.996  (0.992-1.000) | 0.060 | 0.997  (0.992-1.001) | 0.170 | 0.997  (0.992-1.002) | 0.199 |
| Q1 |  |  |  |  |  |  |  |  |  |  |  |  |
| Q2 | 0.654  (0.462-0.924) | **0.016** | 0.628  (0.431-0.915) | **0.015** | 0.634  (0.434-0.927) | **0.019** | 0.771  (0.482-1.232) | 0.277 | 0.804  (0.487-1.326) | 0.393 | 0.957  (0.562-1.630) | 0.872 |
| Q3 | 0.572  (0.403-0.811) | **0.002** | 0.535  (0.366-0.783) | **0.001** | 0.548  (0.373-0.804) | **0.002** | 0.276  (0.144-0.531) | **＜0.001** | 0.257  (0.129-0.510) | **＜0.001** | 0.240  (0.114-0.507) | **＜0.001** |
| Q4 | 0.458  (0.323-0.649) | **＜0.001** | 0.489  (0.334-0.717) | **＜0.001** | 0.524  (0.354-0.775) | **0.001** | 0.697  (0.422-1.150) | 0.158 | 0.774  (0.453-1.322) | 0.348 | 0.811  (0.452-1.455) | 0.482 |
| **TygI** | 3.043  (1.731-5.350) | **＜0.001** | 2.452  (1.295-4.643) | **0.006** | 2.268  (1.194-4.309) | **0.012** | 0.603  (0.277-1.312) | 0.202 | 0.454  (0.194-1.063) | 0.069 | 0.481  (0.190-1.214) | 0.121 |
| **Fib-4** | 0.786  (0.676-0.913) | **0.002** | 0.853  (0.723-1.007) | 0.060 | 0.932  (0.774-1.123) | 0.460 | 1.932  (1.584-2.358) | **＜0.001** | 2.604  (2.023-3.352) | **＜0.001** | 2.102  (1.580-2.796) | **＜0.001** |
| Without Diatebes |  |  |  |  |  |  |  |  |  |  |  |  |
| **PLR**  **(Cat)** | 0.772  (0.683-0.873) | **＜0.001** | 0.849  (0.736-0.978) | **0.024** | 0.847  (0.733-0.978) | **0.023** | 0.789  (0.536-1.162) | 0.229 | 0.794  (0.525-1.201) | 0.275 | 0.767  (0.503-1.169) | 0.217 |
| **PLR (Con)** | 0.997  (0.996-0.998) | **＜0.001** | 0.998  (0.997-1.000) | **0.041** | 0.999  (0.997-1.000) | 0.060 | 0.998  (0.994-1.002) | 0.417 | 0.999  (0.994-1.003) | 0.604 | 0.999  (0.994-1.003) | 0.580 |
| Q1 |  |  |  |  |  |  |  |  |  |  |  |  |
| Q2 | 0.869  (0.733-1.031) | 0.107 | 0.847  (0.696-1.031) | 0.097 | 0.875  (0.718-1.067) | 0.186 | 0.504  (0.292-0.872) | **0.014** | 0.443  (0.250-0.784) | **0.005** | 0.473  (0.265-0.846) | **0.012** |
| Q3 | 0.772  (0.651-0.915) | **0.003** | 0.795  (0.653-0.966) | **0.021** | 0.794  (0.652-0.968) | **0.023** | 0.768  (0.474-1.432) | 0.282 | 0.739  (0.444-1.230) | 0.245 | 0.732  (0.434-1.235) | 0.242 |
| Q4 | 0.652  (0.548-0.774) | **＜0.001** | 0.753  (0.617-0.918) | **0.005** | 0.777  (0.635-0.951) | **0.014** | 0.559  (0.331-0.944) | **0.030** | 0.545  (0.310-0.956) | **0.034** | 0.550  (0.310-0.976) | **0.041** |
| **TygI** | 3.956  (3.378-4.632) | **＜0.001** | 2.636  (2.201-3.158) | **＜0.001** | 2.469  (2.057-2.964) | **＜0.001** | 1.860  (1.152-3.002) | **0.011** | 1.142  (0.672-1.940) | 0.624 | 1.035  (0.601-1.780) | 0.902 |
| **Fib-4** | 1.009  (0.943-1.079) | 0.801 | 1.174  (1.063-1.297) | **0.002** | 1.148  (1.027-1.283) | **0.015** | 1.312  (1.073-1.605) | **0.008** | 1.591  (1.299-1.948) | **＜0.001** | 1.207  (1.034-1.409) | **0.017** |

Model1: No covariates adjusted;

Model2: Gender,Race,BMI,Sleeping time,Smoking status adjusted;

Model3: Gender,Race,BMI,C-Reactive Protein,Albumin,ALP,GGT,LDH,Blood Urea Nitrogen,Sleeping time,Smoking status adjusted.
